# The Efficacy of Metacognitive Therapy: A Systematic Review and Meta-Analysis

**DOI:** 10.3389/fpsyg.2018.02211

**Published:** 2018-11-14

**Authors:** Nicoline Normann, Nexhmedin Morina

**Affiliations:** ^1^Department of Psychology, University of Copenhagen, Copenhagen, Denmark; ^2^Department of Clinical Psychology and Psychotherapy, Institute of Psychology, University of Münster, Münster, Germany

**Keywords:** metacognitive therapy, meta-analysis, psychotherapy, anxiety, depression, psychopathology, mental disorders

## Abstract

**Background:** Metacognitive therapy (MCT) continues to gain increased ground as a treatment for psychological complaints. During the last years, several clinical trials on the efficacy of MCT have been published. The aim of the current study was to provide an updated meta-analytic review of the effect of MCT for psychological complaints.

**Methods:** We conducted a systematic search of trials on MCT for young and adult patients with psychological complaints published until January 2018, using PsycINFO, PubMed, the Cochrane Library, and Google Scholar. Trials with a minimum of 10 participants in the MCT condition were included.

**Results:** A total of 25 studies that examined a variety of psychological complaints met our inclusion criteria, of which 15 were randomized controlled trials. We identified only one trial that was conducted with children and adolescents. In trials with adult patients, large uncontrolled effect size estimates from pre- to post-treatment and follow-up suggest that MCT is effective at reducing symptoms of the targeted primary complaints, anxiety, depression, and dysfunctional metacognitions. The comparison with waitlist control conditions also resulted in a large effect (Hedges' *g* = 2.06). The comparison of MCT to cognitive and behavioral interventions at post-treatment and at follow-up showed pooled effect sizes (Hedges' g) of 0.69 and 0.37 at post-treatment (*k* = 8) and follow-up (*k* = 7), respectively.

**Conclusions:** Our findings indicate that MCT is an effective treatment for a range of psychological complaints. To date, strongest evidence exists for anxiety and depression. Current results suggest that MCT may be superior to other psychotherapies, including cognitive behavioral interventions. However, more trials with larger number of participants are needed in order to draw firm conclusions.

Metacognitive therapy (MCT; Wells, 2009) continues to gain ground as a treatment for psychological complaints. MCT is theoretically grounded in the self-regulatory executive function model (Wells and Matthews, 1994, 1996), which states that psychopathology arises as a result of a perseverative thinking style called the cognitive attentional syndrome (CAS). The CAS consists of dysfunctional coping strategies that a person employs as an attempt to manage distressful thoughts and feelings. These include worry, rumination, threat monitoring, thought control strategies, avoidance, and reassurance seeking (Wells, 2009). The model proposes that negative thoughts and feelings are temporary in nature, however, when a person responds to these with CAS activity, this may cause extended psychological distress and may inadvertently exacerbate and prolong negative affect. The model further suggests that the CAS arises from a person's positive and negative metacognitive beliefs, i.e., beliefs about cognition. Positive metacognitions are beliefs about the need to engage in CAS activities, e.g., “Worry helps me stay prepared,” whereas negative metacognitions are beliefs about the uncontrollability and dangerousness of thoughts and feelings, e.g., “I have no control over my worry/rumination” and “Feeling like this means I am losing my mind” (Wells, 2009).

In MCT, metacognitive beliefs and processes related to the CAS are identified and modified during treatment. The treatment is manualized, as outlined by Wells (2009). However, flexible application of the manuals is advocated to fit the specific patient's needs. Although MCT targets transdiagnostic processes, the exact case formulation model as well as combination of techniques vary depending on the disorder in question. The first step in therapy is to conceptualize an idiosyncratic case formulation together with the patient, and to socialize the patient to the maintaining processes, including the impact of worry and rumination and the ineffectiveness of current coping strategies. Next, metacognitive beliefs are verbally challenged in Socratic dialogues, and behavioral experiments are used to test and generate change in the person's metacognitive predictions or beliefs about CAS strategies. Main emphasis is laid on challenging the negative beliefs before moving on to challenging the positive metacognitive beliefs. The patient is instructed to postpone worry and rumination processes. The aim is for patient to experience that worry and rumination are processes that can be postponed by disengaging from further processing, that they are harmless, and have no advantages. Specifically designed therapeutic techniques, such as the attention training technique or detached mindfulness (Wells, 2009), are used. The attention training technique (Wells, 1990) is an auditory task that requires the patient to engage in selective attention, divided attention, and attention switching. It is designed to increase the patient's executive control and regain attentional flexibility. In detached mindfulness the patient is instructed to become aware of internal trigger thoughts and detach from them by taking a step back and disengaging any further coping or perseverative processing in reaction to them. The patient practices these new ways of reacting to trigger thoughts in therapy as well as between sessions, and their implementation is proposed to strengthen the patient's ability to disengage from worry and rumination processes. The techniques furthermore challenge the patient's belief that worry and rumination are uncontrollable. Toward the end of therapy focus is on reversing any residual CAS activity. Altogether, MCT aims at increasing the person's experience of attentional control, reducing self-focused attention, and fostering the development of adaptive beliefs and coping strategies.

Several clinical trials have examined the efficacy of MCT. Normann et al. (2014) meta-analytically summarized relevant trials on MCT that were published until early 2014. The authors incorporated 16 trials with patients with anxiety and depression and concluded that MCT is very effective in these populations. It must be noted, however, that only nine of the trials in this meta-analysis were controlled trials and most trials were based on rather small samples. Very recently, Rochat et al. (2018) assessed the efficacy of single-case studies on MCT in a meta-analytic review and also reported that these studies support treatment efficacy of MCT for anxiety, depression, and other psychopathological symptoms. Since the meta-analysis by Normann et al. (2014), several clinical trials on the effect of MCT have been published. Furthermore, the meta-analysis by Normann et al. (2014) focused on depression and anxiety disorders only. To address these limitations, the current study aimed at providing an updated review and meta-analysis on the effect of MCT. The main objective was to investigate whether MCT improves symptoms of psychological complaints on primary and secondary outcome variables in comparison to control conditions. For this purpose, we focused on both uncontrolled as well as controlled trials. With regard to the secondary outcomes, we aimed at assessing whether treatment has an impact on comorbid anxiety or depression as well as metacognitions.

## Methods

The aims and methods of this meta-analysis have been registered with the International Prospective Register for Systematic Reviews, with ID number CRD42018084507 (available from https://www.crd.york.ac.uk/PROSPERO). The meta-analysis was conducted using the guidelines and checklist outlined by the Preferred Reporting Items for Systematic reviews and Meta-Analyses (PRISMA) Group (Moher et al., 2009). Accordingly, our main research question describing the Population, Intervention, Comparison, Outcome, and Study design (PICOS) was: In individuals with psychological complaints (P), does metacognitive therapy (I), compared to control conditions (C), improve symptoms of psychopathology (O) in randomized controlled trials (S)? However, due to the limited number of controlled trials meeting our criteria, we decided to also include uncontrolled trials and thus first examine the efficacy of treatment with respect to within-group effect sizes (i.e., change from pre- to post-treatment or follow-up). We first examined the efficacy of treatment from pre- to post-assessment for the primary outcome of all included studies. However, since uncontrolled effect sizes do not account for the impact of time on symptoms, we view controlled effect sizes as more reliable when it comes to assessing treatment efficacy. We also calculated effect sizes for secondary outcome measures of anxiety, depression and metacognitions to the extent these were available. With respect to between-group analyses, we calculated effect sizes for the primary outcome on studies comparing MCT with waitlist control and active treatment control conditions, respectively.

### Eligibility criteria

The criteria for inclusion of a trial in the meta-analysis were: The study had to (1) evaluate MCT as developed by Adrian Wells, and (2) have a sample size of at least 10 patients with psychological complaints in the MCT condition. In order to include as many trials as possible, we did not place any a priori restrictions on study design, comparison conditions, age of participants, publication type, or statistical presentation of results. We excluded studies that examined specific MCT techniques in isolation (e.g., attention training) as opposed to the treatment as a whole, and studies that combined MCT techniques with other types of therapy, e.g., cognitive therapy. Studies had to be written in English, Danish, Norwegian, Swedish, Dutch, or German in order to be included, as these were the languages at least one of the authors is proficient in.

### Literature search

As this study is an update of a previous meta-analysis (Normann et al., 2014), trials that were included in the previous meta-analysis were also included in this meta-analysis if they fulfilled our current inclusion criteria. We identified additional studies by searching the databases PsycINFO, PubMed, and the Cochrane Library for the period between January 2014 and January 2018, using the search string “(metacognitive or meta-cognitive) AND (therapy OR trial OR treatment OR psychotherap* OR intervention).” We also conducted a backward search of the reference lists from articles that met the inclusion criteria. Further, trial registries (www.clinicaltrials.gov; www.isrctn.com) were searched for potential completed trials on MCT. Google Scholar was included as an additional information source. In Google Scholar the search string was limited to articles that used the term “metacognitive therapy” in their title, as the full search string yielded more than 1,000 hits, which is more than the database was able to display. The last search was conducted January 11th, 2018.

### Study selection and data extraction

After removing duplicates, the titles and abstracts of all search hits were screened, and those that did not fulfill our inclusion criteria were excluded. The full text versions of the remaining records were retrieved and assessed for eligibility for inclusion in the meta-analysis. The final list of studies was jointly discussed by both authors.

For each included publication, a list of study characteristics was extracted: type of psychological complaint treated, comparison conditions, sample size, attrition rates at the end of therapy, gender distribution, mean age, comorbidity rates, number of therapy sessions, intervention format (individual or group), follow-up period(s) from the end of therapy, statistical analyses (completer or intent-to-treat), and treatment fidelity checks. We also extracted information for the effect size calculation, namely means and standard deviations for the primary and secondary outcome measures at pretreatment, post-treatment and the longest reported follow-up period available. Data from intent-to-treat samples was used to the extent possible. The primary outcome measure was chosen based on which measure had the most specific relevance for the psychological complaint in question. If a study did not provide sufficient data for performing the meta-analysis, or if central study characteristics were lacking, the information was requested from the authors of the study in question via e-mail. Study selection and extraction of study characteristics was performed by the first author, in consultation with the second author.

### Risk of bias assessment

We assessed the quality of reporting of each included study with the Risk of Bias tool developed by the Cochrane Collaboration (Higgins et al., 2011). The risk of bias of the individual studies was examined across six domains: random sequence generation, allocation concealment, blinding of outcome assessment, incomplete outcome data, selective reporting, and other sources of bias. Each domain was assigned a judgement of low, unclear, or high risk of bias. An “unclear” judgement was given if the reporting of what happened in the study was not described in suffice detail to allow judgement of either low or high risk of bias. We did not assess the blinding of participants and personnel (performance bias), as it is not feasible to blind therapists and clients to a psychotherapeutic intervention. In order to ensure consistency in the judgements across all studies, we chose to reassess the risk of bias for the studies from the previous meta-analysis, as those judgements were based on three raters. Four studies were consensus-coded by both authors, and interrater reliability was established for the remaining studies, which were all double-coded. The intraclass correlation coefficient using a two-way random effects model (absolute agreement; single measurement) was 0.84, 95% CI [0.78–0.89], indicating very good reliability. Subsequently, discrepancies in the codes were handled through discussion, until consensus was reached.

### Statistical analyses plan

The meta-analysis was carried out using the software program Comprehensive Meta-Analysis (version 3.3; Borenstein et al., 2009). Due to the heterogeneity of the included studies, we expected there to be natural variations in the distribution of the true effect sizes, and therefore a random effects model was used (Borenstein et al., 2009). Hedges' *g* was chosen as the effect size metric throughout the meta-analysis. Similarly to Cohen's *d*, it is based on the standardized mean difference, but it applies a correction factor to obtain an unbiased estimate in small samples (Borenstein et al., 2009). Values of 0.2, 0.5, and 0.8 can be conservatively interpreted as small, medium, and large magnitudes of effect, respectively (Cohen, 1988). The correlation between the measures at pre- and post-treatment and pretreatment and follow-up was needed for the effect size calculation, and this was not provided in the studies. As recommended by Morris and DeShon (2002), we retrieved these correlations from authors in a subset of the studies, and conservatively estimated the correlation to *r* = 0.50, which corresponded to the upper limits of the confidence intervals of the aggregate correlations in the subset of studies.

As mentioned above, we first computed within-group effect sizes (i.e., change from pre- to post-treatment or follow-up) for the primary outcome of all included studies at post-treatment and follow-up. We also calculated effect sizes for secondary outcome measures of anxiety, depression and metacognitions, to the extent these were available. With regard to between-group analyses, we calculated effect sizes for the primary outcome on studies comparing MCT with waitlist control and active treatment control conditions, respectively.

In order to measure variability in the study outcomes, we used the *I*^2^ statistic, which describes the percentage variation across studies that is due to heterogeneity rather than chance. It has been suggested that *I*^2^ values of 25, 50, and 75% may be interpreted as referring to low, moderate, and high levels of heterogeneity (Higgins et al., 2003). We further performed subgroup analyses for subgroups of at least four trials, as has been recommended (Fu et al., 2011). Due to the low number of trials included in these analyses, the *p*-value for statistical significance was set to 0.1.

We also assessed potential publication bias using visual inspection of funnel plots of the primary outcome measures. In accordance with recommendations from Sterne et al. (2011), publication bias was assessed if there was a minimum of 10 studies available. Particularly, we were interested in examining whether there was an asymmetry in the plot with smaller studies having larger effect sizes, which is indicative of publication bias (Sterne et al., 2011). The trim-and-fill procedure by Duval and Tweedie (2000) was used to calculate the likely number of missing studies and estimate an effect size that corrects for publication bias.

## Results

### Search results and study selection

**Figure 1:**
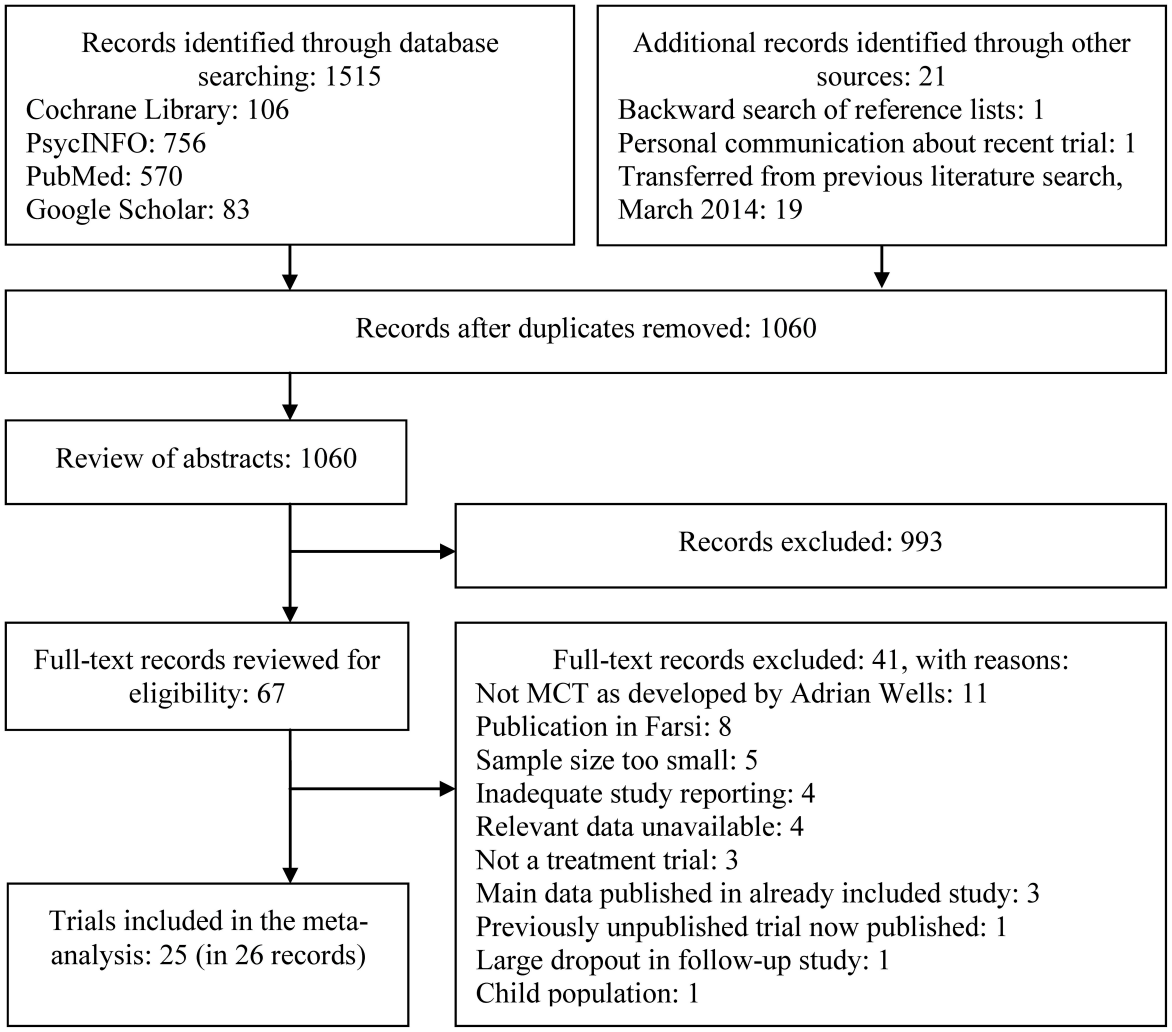
Flow diagram of study selection process.

Figure 1 displays a PRISMA (Moher et al., 2009) flow diagram of the study selection process. A total of 1536 records were identified, and 25 trials were eligible for inclusion in the final meta-analysis. Eleven of the eligible studies were from the 2014 search (of which nine were included in the analyses), whereas the additional 14 studies were identified through the updated search. Two of these (Wells et al., 2015; Nordahl et al., 2018) were peer-reviewed publications of trials that were included in the first meta-analysis, which at that time only were available as dissertations. With the exception of one study (Esbjørn et al., 2018), all other studies that fulfilled our inclusion criteria were conducted with adult populations. Our results therefore focus on the efficacy of MCT for adults only.

### Study characteristics

Table 1 provides an overview of the studies included in the meta-analysis, along with their study characteristics. We included 25 trials based on 26 records. Of these, 10 studies compared MCT with active control conditions, 9 compared MCT with waitlist control conditions, and 10 were uncontrolled trials. Eight of the active control conditions were cognitive and/or behavioral interventions. These included generic and disorder-specific cognitive behavioral therapy (*k* = 5), behavioral activation (*k* = 1), applied relaxation (*k* = 1), and prolonged exposure (*k* = 1). Other interventions included mindfulness-based stress reduction (*k* = 1) and Masters-Johnson sex therapy (*k* = 1).

**Table 1:** Study characteristics.

**Psychological complaint**	**Study and treatment type**	**Primary outcome**	**Treatment format**	***n* analyzed**	**% attrition**	**Flw-up months**	**% female**	**Mean age**	**Comorbidity**	**Therapy sessions**	**Statistical analysis**
**DEPRESSIVE DISORDERS**											
MDD	Dammen et al., 2015, 2016										
	MCT	BDI	Group	11	0	24	100	42.3	91%	11.5	(ITT)^a^
MDD	Hagen et al., 2017										
	MCT	BDI	Individual	39	5.4	6	59	33.7	67%^b^	10	ITT
	WL			19	10.5		53	35.4			
MDD	Hjemdal et al., 2017										
	MCT	BDI	Individual	10	0	6	80	28.4	100%	10	(ITT)
Depressive disorders	Jordan et al., 2014										
	MCT	QUIDS_16_-C	Individual	23	21.7	6	48	37.2	Minimum 74%^c^	11.5^d^	ITT
	CBT		Individual	25	24		48	35.0	Minimum 60%^c^	11.5^d^	
MDD	Papageorgiou and Wells, 2015										
	MCT	BDI	Group	10	0	6	80	41.7	90%	14	(ITT)
MDD	Shareh and Dolatshahi, 2012										
	MCT	BDI	Group	10	16.7	–	80	NI	NI	8	Compl
	WL			10	16.7		60	NI	NI		
MDD	Wells et al., 2012										
	MCT	BDI	Individual	12	16.7	12	92	34.5	75%	6.5	ITT
MDD	Zemestani et al., 2016										
	MCT	BDI	Group	15	0	3	61 ^b^	24.2^b^	19 diagnoses on *n* 45^b^	8	(ITT)
	BA		Group	15	0					8	
	WL			15	0						
**GENERALIZED ANXIETY DISORDER**											
	Nordahl et al., 2018										
	MCT	PSWQ	Individual	32	0	24	75	37.0	44 diagnoses	12	ITT
	CBT		Individual	28	0		68	38.6	41 diagnoses	12	
	WL			21	0		76	37.9	35 diagnoses		
	van der Heiden et al., 2012										
	MCT	PSWQ	Individual	61	18.0	6	70	33.9	59%	12.3	ITT
	IUT		Individual	60	23.3		69	34.4	60%	12.9	
	WL			20	5.0		90	39.6	75%		
	van der Heiden et al., 2013										
	MCT	PSWQ	Group	33	27.3	(6)	64	31.3	73%	12.9	ITT
	Wells and King, 2006										
	MCT	STAI-T	Individual	8 (10)^e^	0	12	60	(25-75)	50%	7.4	(ITT)
	Wells et al., 2010										
	MCT	PSWQ	Individual	10	0	12	60^b^	49.1^b^	80%^b^	12	(ITT)
	AR		Individual	10	0					12	
**POST-TRAUMATIC STRESS DISORDER**											
	Wells et al., 2008										
	MCT	IES	Individual	11	15.4	6	55	38.9	55%	8.5	Compl
	Wells and Colbear, 2012										
	MCT	PDS	Individual	10	10	6	60	33.4	8 diagnoses	6.4	ITT
	WL			10	0		50	41.3	6 diagnoses		
	Wells et al., 2015										
	MCT	PDS	Individual	10	9.1	3	36	40.6	46%	8	Compl
	PE		Individual	10	9.1		36	40.5	55%	8	
	WL			10	0		40	42.7	70%		
**TRANSDIAGNOSTIC SAMPLE**											
Anxiety and depression	Capobianco et al., 2018										
	MCT	HADS	Group	17	17.6	6	72	30.4	NI	8	ITT
	MBSR		Group	18	27.8		70	26.7	NI	8	
Anxiety	Johnson et al., 2017										
	MCT	BAI	Individual	36	5.6	12	61^b^	42.0^b^	91%^b^	9.4	ITT^f^
	CBT (disorder specific)		Individual	38	13.2					9.4	
Mixed disorders	Nordahl, 2009										
	MCT	BAI	Individual	15	0	-	60	37.2	31 total diagnoses	7.5	Compl
	CBT		Individual	13	13.3		62	34.9	26 total diagnoses	10.2	
**OTHER PSYCHOLOGICAL COMPLAINTS**											
Cancer distress	Fisher et al., 2015										
	MCT	HADS	Individual	12	16.7	6	33	20.7	NI	7.8	ITT
Schizophrenia	Morrison et al., 2014										
	MCT	PANSS	Individual	10	20	3	20	34.3	NI	10.6	ITT
Body dysmorphic disorder	Rabiei et al., 2012										
	MCT	BDD-YBOCS	Individual	10	0	6	90^b^	23.7	40%	8	(ITT)
	WL			10	0			26.6	50%		
Hyposexual. desire disorder	Ramezani et al., 2017										
	MCT	FSFI	Individual	15	NI	6	80	32.1	0%	10	Compl
	MJST		Individual	15	NI		73	33.3	0%	10	
Obsessive compulsive disorder	van der Heiden et al., 2016										
	MCT	Y-BOCS	Individual	25	24	3	68	32.3	52%	13.7	ITT
Grief	Wenn, 2017										
	MCT	PG-13	Group	21	21.1	6	95^b^	62	Minimum 64%^c^	6	ITT
	WL			10	10			62	Minimum 50%^c^		

*Percent attrition is at post-treatment. Follow-up months indicates the longest follow-up period from post-treatment, and parenthesis indicates that the follow-up was not used in the analyses. Means are given for number of therapy sessions, and if means are not available, the maximum number of sessions allowed is stated. N analyzed refers to number of participants that data was available for*.

a *Follow-up analyses did not use ITT*.

b *Refers to the total sample, as data was not available for each group*.

c *Comorbid anxiety disorders*.

d *Median number of sessions*.

e *8 analyzed for primary outcome, 10 for secondary outcomes*.

f *MCQ data was based on completers. AR, applied relaxation; BA, behavioral activation; BAI, Beck Anxiety Inventory; BDD-YBOCS, Yale-Brown Obsessive-Compulsive Scale Modified for Body Dysmorphic Disorder; BDI, Beck Depression Inventory; CBT, cognitive behavior therapy; Compl, completer analysis; FSFI, Female Sexual Function Index; HADS, Hospital Anxiety and Depression Scale; IES, Impact of Events Scale; ITT, intention-to-treat analysis; (ITT), no attrition, thus equivalent to intention-to-treat analysis; IUT, intolerance-of-uncertainty therapy; MBSR, mindfulness based stress reduction; MDD, major depressive disorder; MJST, Masters-Johnson Sex Therapy; NI, No information; PANSS, Positive and Negative Syndromes Scale; PDS, Post-traumatic Stress Diagnostic Scale; PE, prolonged exposure; PG13, Prolonged Grief Disorder Scale; PSW Q, Penn State Worry Questionnaire; QUIDS_16_-C, Quick Inventory of Depressive Symptomatology-Clinician rating; STAI-T, State-Trait Anxiety Inventory—Trait scale; WL, waitlist; Y-BOCS, Yale-Brown Obsessive-Compulsive Scale*.

The trials were conducted in the United Kingdom (*k* = 10), Norway (*k* = 6), Iran (*k* = 4), the Netherlands (*k* = 3), Australia (*k* = 1) and New Zealand (*k* = 1). One study was a PhD dissertation (Wenn, 2017), one was an unpublished manuscript (Shareh and Dolatshahi, 2012), and the remaining 24 records were articles published in peer-reviewed journals.

### Patient characteristics

A large proportion of the identified studies treated patients suffering from anxiety and depression (see Table 1). There were eight trials on depressive disorders. Of these, seven were on major depressive disorder, whereas one study also included a small proportion of patients with bipolar II and bipolar not-otherwise-specified (Jordan et al., 2014). Five trials were conducted on generalized anxiety disorder, three were conducted on post-traumatic stress disorder, and three were conducted on transdiagnostic samples with anxiety and/or depression. The remaining six trials were on cancer distress, schizophrenia spectrum disorders, body dysmorphic disorder, hyposexual desire disorder, obsessive-compulsive disorder, and grief.

The majority of studies included participants that fulfilled criteria for a psychological disorder according to either DSM-IV-TR (American Psychiatric Association, 2000) or ICD-10 (World Health Organization, 1992) criteria. Two studies (Fisher et al., 2015; Capobianco et al., 2018) did not use structured psychiatric interviews, but rather included patients with elevated levels of anxiety and/or depression based on a cut-off score from a self-report. One study (Wenn, 2017) assessed diagnostic criteria, but also included participants who did not meet the criteria in question. With few exceptions, the reported comorbidity rates were high. Four studies did not report comorbidity rates, and one study (Ramezani et al., 2017) excluded patients with comorbid disorders. For the 17 studies that reported comorbidity rates in percentages, the mean for the MCT conditions was 65% (standard deviation 24, range 0–100%). These were primarily Axis I disorders consisting of anxiety and depressive disorders. Few studies also reported relatively low rates of substance abuse and eating disorders. Further, inclusion of patients with certain Axis II disorders was reported in seven studies, with reported rates ranging between 8.3 and 50%. Seven studies specified that they worked with refractory cases (ranging from 25 to 100% of participants) that had not previously responded to other forms of psychotherapy. One study was conducted on an inpatient group (Johnson et al., 2017), whereas the remaining studies were conducted in outpatient settings. All studies used adult samples, with the exception of one study that also included adolescents from age 16 and up (Rabiei et al., 2012).

Altogether, 780 patients were included in the meta-analysis. Of these, 468 were offered MCT and meta-analyzed at post-test. In the post-test comparisons with waitlist controls, 208 patients were in the MCT condition and 125 were in the control condition. Data from control patients that received treatment after their waiting period was included and thus meta-analyzed twice in separate groups. In the post-test comparison with active treatment controls, 234 patients were in the MCT condition and 232 were in the control condition. The mean number of participants included in each trial was 31.2 (standard deviation 27.3, range 10–126).

### Metacognitive therapy

Individual therapy was applied in 18 of the trials, whereas a group format was applied in seven studies. The vast majority of studies (*n* = 18) followed a published disorder-specific treatment manual for the primary disorder, whereas four studies followed the generic model of intervention as presented by Wells (2009). Three studies (Rabiei et al., 2012; Morrison et al., 2014; Ramezani et al., 2017) investigated a psychological disorder for which no formal manual had yet been developed. In these cases, the authors had adapted a treatment manual for another disorder to the disorder in question. Number of mean therapy sessions ranged from 6 to 14, with an overall mean of 9.5 (standard deviation 2.3) across all 25 studies. Group sessions tended to last between 90 and 120 min, whereas individual sessions usually were between 45 and 60 min. Typically, MCT was conducted weekly. However, some trials chose to intensify treatment in the beginning of therapy, and others chose to prolong treatment as to allow for incorporation of techniques into everyday life. For example, the study on schizophrenia delivered 12 sessions over approximately 9 months (Morrison et al., 2014). With regard to treatment fidelity, the majority (*n* = 19) of studies reported that continued supervision was provided from experts in order to ensure adherence to the treatment protocols. However, only six studies had assessed treatment fidelity in a formal manner, i.e., with checklists and video or audio recordings, and conclude that therapists adequately adhered to the protocols.

### Outcome measures

The primary outcome measure of each study is presented in Table 1. With regard to the secondary outcome measures, in the majority of cases the Beck Anxiety Inventory (Beck et al., 1988) and Beck Depression Inventory(-II) (Beck et al., 1961, 1996) were used as measures of anxiety and depression symptoms, respectively. With respect to measures of metacognition, the Metacognitions Questionnaire(-30) (Cartwright-Hatton and Wells, 1997; Wells and Cartwright-Hatton, 2004), the Positive Beliefs about Rumination Scale (Papageorgiou and Wells, 2001) and the Negative Beliefs about Rumination Scale (Papageorgiou et al., 2003) were mostly used. Thirteen of the publications reported on the efficacy of treatment on positive and negative metacognitions separately, whereas five publications reported on the efficacy of MCT on metacognitions in general, without distinguishing between positive and negative metacognitions. We conducted the analyses accordingly. The measures included in each of the secondary analyses are listed in Table 3.

### Follow-up

Out of the 25 studies, 22 had follow-up data that was included in our analyses. The mean length of the included follow-up periods was 8.2 months from post-treatment (standard deviation 5.9, range 3–24 months). As displayed in Figure 1, we excluded one publication (van der Heiden and Melchior, 2014), which was a 30-month follow-up of an included trial (van der Heiden et al., 2012), as the publication reported on 34 out of the original 126 participants. We further chose not to include the follow-up data in another study (van der Heiden et al., 2013), as the authors had not included the data in their primary analysis due to a large dropout rate.

### Risk of bias

Table 2 presents the risk of bias of each included study. Overall, the most prevalent rating given was low risk of bias. However, unclear risks of bias were present with regard to allocation concealment, as only 5 out of the 15 controlled trials had described in adequate detail how the randomization schedule was concealed, so that participants and assessors could not foresee which treatment they were allocated to. Separating the randomization from the recruitment process is essential for ensuring that researchers or assessors do not influence assignment of potential participants to treatment arms. Of the 25 studies, 18 had an unclear risk of selective reporting, as they did not report whether they had published a study protocol for the study. We found high risks of attrition bias in five studies, where intent-to-treat analyses were not applied. Furthermore, we found high risks of detection bias in two studies, as they had not blinded the outcome assessor for the primary outcome measure at post-treatment. Altogether, the risk of bias was rated as low in 73% of cases, unclear in 23% of the cases, and high in 4% of the cases. Furthermore, the trials did not differ substantially on risk of bias and thus this variable could not be included in subanalyses.

**Table 2:** Risk of bias.

	**Selection bias**		**Detection bias**	**Attrition bias**	**Reporting bias**	**Other bias**
**Study**	**Random sequence generation**	**Allocation concealment**	**Blinding of outcome assessment**	**Incomplete outcome data**	**Selective reporting**	**Other sources of bias**
Capobianco et al., 2018	Low	Low	Low	Low	Low	Low
Dammen et al., 2015, 2016	–	–	Low	High	Unclear	Low
Johnson et al., 2017	Low	Unclear	Low	Low	Low	Low
Jordan et al., 2014	Low	Low	High	Low	Low	Low
Fisher et al., 2015	–	–	Low	Low	Unclear	Low
Hagen et al., 2017	Low	Unclear	Low	Low	Low	Low
Hjemdal et al., 2017	–	–	Low	Low	Unclear	Low
Morrison et al., 2014	–	–	Low	Low	Unclear	Low
Nordahl, 2009	Low	Low	Low	High	Unclear	Low
Nordahl et al., 2018	Low	Unclear	Low	Low	Low	Low
Papageorgiou and Wells, 2015	–	–	Low	Low	Unclear	Low
Rabiei et al., 2012	Unclear	Unclear	High	Low	Unclear	Low
Ramezani et al., 2017	Low	Unclear	Low	Unclear	Unclear	Low
Shareh and Dolatshahi, 2012	Unclear	Unclear	Low	High	Unclear	Low
van der Heiden et al., 2012	Low	Unclear	Low	Low	Unclear	Low
van der Heiden et al., 2013	–	–	Low	Low	Unclear	Low
van der Heiden et al., 2016	–	–	Low	Low	Unclear	Low
Wells and King, 2006	–	–	Low	High	Unclear	Low
Wells et al., 2008	–	–	Low	High	Unclear	Low
Wells et al., 2010	Low	Low	Low	Low	Unclear	Low
Wells et al., 2012	–	–	Low	Low	Unclear	Low
Wells and Colbear, 2012	Low	Unclear	Low	Low	Unclear	Low
Wells et al., 2015	Low	Low	Low	Low	Low	Low
Wenn, 2017	Low	Unclear	Low	Low	Low	Low
Zemestani et al., 2016	Low	Unclear	Low	Low	Unclear	Low

### Treatment effects

#### Within-group effect sizes

**Figure 2:**
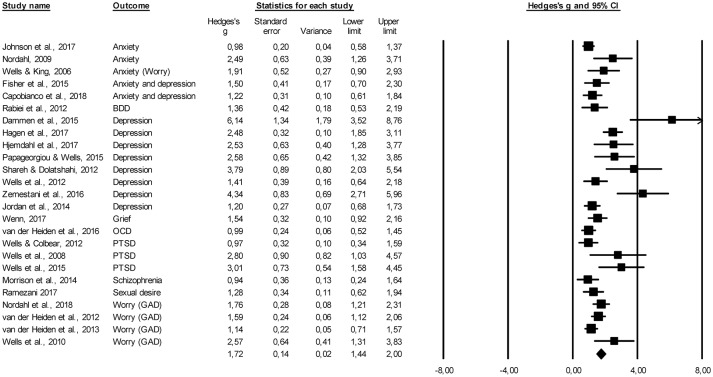
Forest plot of within-group effect size estimates for the efficacy of MCT on primary outcome measures from pre- to post-treatment. BDD, body dysmorphic disorder; GAD, generalized anxiety disorder; OCD, obsessive-compulsive disorder; PTSD, post-traumatic stress disorder.

Figure 2 displays a forest plot of the effect sizes from pre- to post-treatment on the primary outcome measures across all 25 included studies. The pooled pre- to post-treatment effect size was large, *g* = 1.72, 95% CI [1.44–2.00], *p* < 0.001, and this effect was maintained over time, as evidenced by the large pretreatment to follow-up effect size, *g* = 1.57, 95% CI [1.26–1.87], *p* < 0.001, *k* = 22. Subanalyses revealed that MCT also resulted in large and significant reductions of secondary outcome measures that included anxiety, depression, and dysfunctional metacognitions (see Table 3). The pooled effect sizes on the primary outcome measures and for measures of anxiety, depression, and metacognitions are displayed in Table 3.

**Table 3:** Pre- to post-treatment and pretreatment to follow-up effect sizes.

**Domain**	**Post-treatment**				**Follow-up**			
	**Hedges' *g***	**95% CI**	***k***	**Z**	**Hedges' *g***	**95% CI**	***k***	**Z**
Primary outcome	1.72	1.44–2.00	25	12.19	1.57	1.26–1.87	22	10.13
Anxiety as secondary outcome	1.48	1.18–1.78	17	9.64	1.32	1.06–1.58	15	9.95
Depression as secondary outcome	1.12	0.86–1.39	12	8.19	0.97	0.71–1.23	11	7.23
Positive MC	0.86	0.58–1.15	13	5.95	1.02	0.76–1.28	11	7.69
Negative MC	1.31	1.01–1.62	13	8.60	1.28	1.01–1.55	11	9.32
General MC	1.79	0.66–1.70	5	9.57	n.a.			

*k, number of studies included in the analysis; n.a., not applicable, as number of trials too small to conduct analysis; MC, metacognitions. Effect sizes were based on the following questionnaires: For anxiety: Beck Anxiety Inventory; Depression Anxiety Stress Scales-Anxiety; Hospital Anxiety and Depression Scale-Anxiety; State-Trait Anxiety Inventory-Trait. For depression: Beck Depression Inventory; Beck Depression Inventory-II; Depression Anxiety Stress Scales-Depression; Hospital Anxiety and Depression Scale-Depression. For positive metacognitions: Cognitive Attentional Syndrome-1 (positive belief items); Metacognitive Questionnaire (MCQ) or MCQ-30 (positive beliefs subscale); Positive Beliefs about Rumination Scale. For negative metacognitions: Cognitive Attentional Syndrome-1 (negative belief items); Metacognitive Questionnaire (MCQ) or MCQ-30 (negative beliefs about uncontrollability and danger subscale); Negative Beliefs about Rumination Scale. For general metacognitions: Anxious Thoughts Inventory-Meta Worry; Thought Control Questionnaire-Worry; Thought Fusion Inventory*.

#### Between-group effect sizes

**Figure 3:**
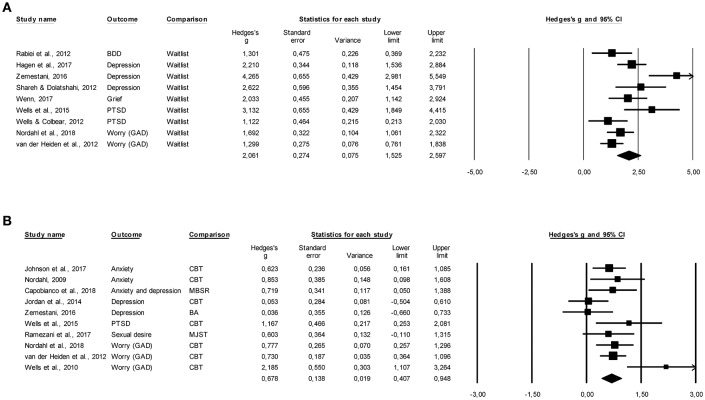
**(A)** Pre- to post-treatment effect sizes and forest plots for MCT compared to waitlist control conditions. **(B)** Pre- to post-treatment effect sizes and forest plots for MCT compared to active control conditions.

Figures 3A,B display the pre- to post-treatment effect sizes and forest plots for MCT compared with waitlist and active control conditions for the primary outcome measures. A large pre- to post-treatment effect size was found for the studies comparing MCT to waitlist controls on primary outcome measures, *g* = 2.06, 95% CI [1.52–2.60], *k* = 9. Only two studies assessed the efficacy of MCT as compared to the waitlist at follow-up, therefore no meta-analytic synthesis was conducted.

Comparison of MCT with active control conditions revealed a medium to large effect size in favor of MCT, *g* = 0.68, 95% CI [0.41–0.95], *k* = 10. This comparison at follow-up revealed a small to medium effect size favoring MCT, *g* = 0.39, 95% CI [0.15–0.63], *k* = 9. Given that eight out of ten active control conditions were cognitive and behavioral interventions (see Table 1), we also focused on the comparison of MCT to these interventions. Compared to behavioral activation and cognitive behavior therapy (CBT), a medium to large effect size was found favoring MCT, *g* = 0.69, 95% CI [0.36–1.03], *k* = 8. Compared to behavioral activation and CBT at follow-up, a small to medium effect size was found favoring MCT, *g* = 0.37, 95% CI [0.07–0.66], *k* = 7.

### Heterogeneity

For the pre- to post-treatment within-group effect size on primary outcome measures *I*^2^ was 69.74%, *Q* = 79.84, *p* = < 0.001, indicating a high degree of variability in the study outcomes. Similarly, high heterogeneity was observed at follow-up (*I*^2^ = 74.63, *Q* = 82.79, *p* = < 0.001). For the comparison of MCT with waitlist and cognitive behavioral interventions, heterogeneity values were also large, *I*^2^ = 71.84%, *Q* = 28.41, *p* = < 0.001 and *I*^2^ = 59.09%, *Q* = 17.11, *p* = < 0.001, respectively. We explored the possible sources of heterogeneity by undertaking subgroup analyses, given that at least four trials could be included in the category of interest.

### Subgroup analyses

The subgroup analyses were undertaken using within-group effect sizes, as no relevant subanalyses could be conducted on between group effect sizes. This was the result of a low number of trials in the categories of interest. For example, none of the disorders were investigated in four or more randomized controlled trials that compared the efficacy of MCT to waitlist or an active control condition.

One of the trials produced an effect size of *g* = 6.14 from pre- to post-treatment (Dammen et al., 2015), which may be considered as an outlier from the pooled mean effect size of *g* = 1.72. When this study was excluded, the pre- to post-treatment and pretreatment to follow-up effect sizes did not change substantially (*g* = 1.66, 95% CI [1.40–1.91] and *g* = 1.51, 95% CI [1.22–1.80], respectively).

With respect to the efficacy of MCT for specific psychological complaints, only two disorders were investigated in four or more trials and thus enabled subanalyses. For trials with patients with depression, a large within-group effect size was obtained, *g* = 2.68, 95% CI [1.85–3.51], *k* = 8. A large effect size was also produced when only the trials with patients with GAD were analyzed at post-treatment, *g* = 1.61, 95% CI [1.23–1.98], *k* = 5.

Because 13 of the trials were co-authored by the originator of MCT (Adrian Wells), the possibility of allegiance bias was examined by comparing the results of these studies with the remaining studies. Both groups of publications revealed large effect sizes, with *g* = 1.98, 95% CI [1.52–2.44] for studies by Wells and colleagues and *g* = 1.49, 95% CI [1.17–1.81] for studies by independent authors. The results indicated that the studies conducted by Wells and colleagues produced significantly higher effect sizes (*p* = 0.09). However, when the above mentioned potential outlier (Dammen et al., 2015) was removed, the effect size of the trials conducted by Wells and colleagues was reduced to *g* = 1.84, 95% CI [1.43–2.24], *k* = 12, and the difference between the groups was no longer significant (*p* = 0.19).

Studies that had applied intent-to-treat analyses, including those without dropouts displayed a significantly lower effect (*g* = 1.60, 95% CI [1.32–1.89], *k* = 20) as opposed to those that based their results on completer analyses (*g* = 2.50, 95% CI [1.52-3.49], *k* = 5), *p* = 0.08.

With regard to treatment format, we found that studies that had applied an individual treatment format had a significantly lower effect size (*g* = 1.57, 95% CI [1.30-1.84], *k* = 18) than the trials with a group format (*g* = 2.45, 95% CI [1.59–3.30], *k* = 7), *p* = 0.06. However, when the above mentioned potential outlier (Dammen et al., 2015) was removed, the effect size of the trials applying a group format was reduced to *g* = 2.09, 95% CI [1.34–2.84], *k* = 6, and the difference between the groups was no longer significant (*p* = 0.20). Finally, meta-regressions indicated that pre- to post-treatment changes in positive or negative metacognitions did not significantly explain heterogeneity (*Q* = 0.91, *p* = 0.34 for positive metacognitions and *Q* = 0.59, *p* = 0.44 for negative metacognitions).

### Publication bias

Inspection of the funnel plot depicting the within-group pre- to post-treatment effect sizes for the primary outcome measures revealed an asymmetry indicative of potential publication bias, as the direction of the effect of the smaller trials was toward the right side of the plot, i.e., toward higher effect sizes. Duval and Tweedie's (2000) trim-and-fill procedure identified six studies to be missing, and the produced imputed point estimate resulting from the analysis was *g* = 1.49, 95% CI [1.19–1.79]. Accordingly, the effect size was still large. A pattern of asymmetry was also observed when trials comparing MCT to active control conditions at pre- to post-treatment were examined. Here, Duval and Tweedie's (2000) trim-and-fill procedure identified three studies to be missing, and the produced imputed point estimate resulting from the analysis was *g* = 0.53, 95% CI [0.24; 0.82]. Given that fewer than 10 trials compared MCT to a waitlist, publication bias could not be assessed in this regard.

## Discussion

In this meta-analysis, we set out to investigate whether MCT improves symptoms of psychological complaints on primary and secondary outcome variables in comparison to control conditions. We were able to assess the efficacy of 25 trials on MCT for a variety of psychological complaints, altogether examining 780 adult patients. Due to the relatively low number of studies, we computed both within- and between-group effect sizes. Our results indicate that MCT is effective in alleviating psychological symptomatology as well as maladaptive metacognitions. The results further suggest that MCT is superior to waitlist and active treatment control conditions.

We were able to include 16 trials that were not part of the first meta-analysis on the efficacy of MCT (Normann et al., 2014). In contrast to the first meta-analysis, we included studies on a variety of other psychological complaints, rather than on anxiety and depression only. Despite the stricter inclusion criteria for trials (i.e., a minimum of 10 participants instead of five), the effect sizes found in this meta-analytic update were largely comparable to those found previously (Normann et al., 2014). The within-group analyses yielded overall large effects from pre- to post-treatment across the included trials (*g* = 1.72), and these were maintained at follow-up (*g* = 1.57). Similarly, in nine trials MCT was compared to waitlist control conditions, and large effects were found in favor of MCT (*g* = 2.06). Compared to the last meta-analysis, these results have the advantage of being based on a larger number of studies in each of these groups.

In 10 trials, the efficacy of MCT was compared to a range of other psychotherapeutic interventions. One strength of the included control conditions is that they were evidence-based treatments for the respective disorders. We found that MCT resulted in significantly higher symptom reduction on the primary outcome measures as compared to other therapies, with a medium to large effect size at post-treatment and a small to medium effect size at follow-up. Eight out of 10 of the comparison conditions were forms of cognitive and/or behavioral interventions. When comparing the CBT conditions with MCT, MCT also outperformed CBT at post-treatment and follow-up with a medium to large (*g* = 0.69) and a small to medium (*g* = 0.37) effect size, respectively. This is a slightly lower difference in effect than that previously reported, which was based on five trials and resulted in a large pre- to post-treatment effect size in favor of MCT (*g* = 0.97) (Normann et al., 2014). Although our results indicate that the effect of MCT was significantly higher than in the active control conditions, this is a finding that needs to be interpreted with caution. The number of studies included in these analyses was low and there were variations in the findings across the studies. Furthermore, the difference between MCT and other types of therapy was not as large at follow-up as at post-treatment. This is reflected in the lower bounds of the 95% confidence intervals, which were close to zero in the follow-up comparison. Thus, additional randomized controlled trials with larger sample sizes are needed in order to draw firm conclusions on whether there are differences in treatment effects between MCT and CBT interventions. Furthermore, future research should investigate whether MCT and CBT work differently for different groups of patients with psychological complaints.

MCT was applied to a large variety of psychological complaints. The vast majority of trials, however, targeted anxiety or depression, including post-traumatic stress disorder, as their primary outcome. Accordingly, our results primarily indicate that MCT is effective for alleviating anxiety and depression. The effect of MCT for other psychological complaints, including grief, schizophrenia, body dysmorphic disorder, hyposexual desire disorder, and obsessive compulsive disorder, was only examined in one trial each. With respect to the comparison with CBT, the examined studies exclusively targeted anxiety and depression symptomatology, and therefore they only generalize to this patient group.

We found that MCT not only produced large effects on symptoms related to the targeted problem, but also alleviated secondary, more general symptoms of anxiety and depression. This indicates that MCT also effectively targets comorbid problems of anxiety and depression, which is in line with the theory that MCT targets transdiagnostic processes related to psychopathology (Wells, 2009). This notion relates directly to the finding that MCT produced large changes in metacognitive beliefs and processes at post-treatment and follow-up. In MCT, metacognitions are conceptualized as transdiagnostic beliefs and processes that relate to the development and maintenance of psychological complaints. Visual inspection of the effects for negative metacognitive beliefs indicates that they were larger than for positive metacognitive beliefs. This finding corresponds with the fact that the primary focus in therapy is to challenge negative metacognitive beliefs, as positive metacognitive beliefs about worry and rumination are also prevalent in the general population and less specific to psychopathology (Wells, 2009; Sun et al., 2017). Altogether, these results support the notion that MCT can be effectively applied as a transdiagnostic approach for patients with different psychological disorders. Provided that future empirical data corroborate current results, this would entail great benefits for clinical practice. Effective transdiagnostic approaches enable therapists to more easily conceptualize the common maintaining processes across clinically relevant issues by delivering treatment strategies within the one protocol. This increases not only the efficacy but also the efficiency of treatment as well as the ease of implementation.

Although the pre- to post-treatment effect sizes on the primary outcome measure were within the large range for all trials, the effect produced by the individual studies varied, as indicated by the high degree of heterogeneity. We were able to explore some of the potential reasons for this. One explanation for the heterogeneity is found in the type of statistical analysis used in the studies. As perhaps expected, studies that used completer analyses produced significantly higher effect sizes than those that used intent-to-treat analyses. There was indication that the studies conducted by the originator produced higher effect sizes. It should be noted, however, that the trials conducted by other groups also produced a very large effect size (*g* = 1.49). More importantly, when the study by Dammen et al. (2015) was removed, there was no significant difference between the groups. We found no difference in effect based on treatment format, when the outlying study was removed, suggesting that MCT is equally effective in individual and group formats. Due to the relatively low number of trials, it was not possible to explore other potential reasons for the heterogeneity.

This meta-analysis has both strengths and limitations. One strength compared to the previous meta-analysis on MCT, which had included three trials on depressive disorders, is that we were able to include eight trials on depressive disorders. This enables us to draw stronger conclusions that MCT is an effective therapy for this group of patients. We were also able to examine the effect of MCT for a larger range of disorders than anxiety and depression, which is of relevance, given that meta-analytic findings suggest that dysfunctional metacognitive beliefs and processes are found across psychological disorders (Sun et al., 2017). Furthermore, we were able to more accurately examine long-term treatment effects of MCT. In the first meta-analysis, only eight out of 16 studies provided sufficient information in order to be included in the follow-up analyses, whereas in this meta-analysis 22 out of 25 studies provided follow-up data. Another strength relates to the samples of the included studies. These were samples that were representative of a clinical population, with high rates of comorbidity and previous treatment attempts. One limitation to the current meta-analysis is that we were not able to conduct secondary analyses with use of controlled effect sizes, due to the low number of studies included. This poses the risk of over-estimating the effect sizes, as within-group analyses do not account for changes in symptomatology over time that are not related to the intervention. Notably, however, with regard to the primary outcome measure, we did not find indications that the within-group effect size was over-estimated, as the pooled effect size for MCT compared to waitlist was also large. This highlights the relevance of incorporating open trials, as they continue to provide valuable information on the efficacy of MCT. A further limitation is that risk of bias was unclear or high in almost one third of the cases, and it remains unknown how this may have affected the meta-analytic results. Lastly, we had limited options in assessing allegiance bias. Although the subanalyses of studies conducted by the originator vs. those by other researchers did not show clear indications of allegiance bias, the author groups may still have favored MCT. One noticeable exception was the study by Nordahl et al. (2018), which had a balanced author group with regard to allegiance, as the originators of both the CBT protocol and MCT protocol took part in the study.

Based on the results of this systematic review and meta-analysis, we encourage that future trials on MCT apply randomized control designs with evidence-based comparison conditions, particularly when investigating anxiety or depression, in order to strengthen conclusions on the efficacy of MCT. Based on our assessment of risk of bias in the studies currently available, we recommend that future studies improve the quality of reporting by clarifying how the allocated treatment was concealed from the participant and investigator up until treatment start, and that they publish study protocols prior to running the trials, in order to minimize the risk of bias. Furthermore, results from this meta-analysis underscore the importance of reporting intent-to-treat analyses, in order to not overestimate the treatment effects. Finally, future research needs to examine the efficacy of MCT applied as a transdiagnostic treatment for different clinical populations.

In conclusion, the results of this meta-analysis indicate that MCT is highly effective in reducing symptoms of a range of primary targeted psychological complaints along with symptoms of anxiety, depression, and maladaptive metacognitions. There are preliminary indications that MCT may be more effective than other therapeutic interventions, including cognitive behavioral therapies. However, more studies are needed in order to investigate the accuracy of these preliminary findings.

## Author contributions

NN and NM conceived the study. NN conducted the systematic literature search, screened studies for eligibility, and extracted data from the relevant publications. NM conducted the statistical analyses. NN wrote the first draft of the Introduction, Methods, and Discussion sections of the manuscript. NM wrote the first draft of the Results section and contributed to revisions and modifications of the manuscript. Both authors approved the final version.

### Conflict of interest statement

The authors declare that the research was conducted in the absence of any commercial or financial relationships that could be construed as a potential conflict of interest.
